# Lycium Barbarum Polysaccharide Antagonizes Cardiomyocyte Pyroptosis by Inhibiting the Nrf2/NLRP3 Signal Pathway Against Myocardial Ischemia–Reperfusion Injury

**DOI:** 10.3390/ijms27073198

**Published:** 2026-03-31

**Authors:** Liuxin Wu, Peng Lin, Xiaomeng Yin, Lin Yang, Chunyan Ma, Shulin Wu, Moyan Yang, Guangyuan Yang, Mingyuan Liu

**Affiliations:** 1 School of Pharmaceutical Sciences, Jiamusi University, Jiamusi 154003, China; wulx2023@163.com (L.W.); coco460@163.com (P.L.); yxm13843754512@163.com (X.Y.); yangmyan1023@163.com (M.Y.); 2 School of Basic Medical Sciences, Jiamusi University, Jiamusi 154003, China; yl18845423977@163.com (L.Y.); mcy1174405914@163.com (C.M.); 15765346687@163.com (S.W.)

**Keywords:** *Lycium barbarum* polysaccharide, myocardial ischemia reperfusion injury, pyroptosis, Nrf2/NLRP3 signal pathway, NLRP3 inflammasome, oxidative stress

## Abstract

Myocardial ischemia–reperfusion injury (MIRI) significantly compromises the clinical benefits of revascularization and constitutes a central pathological mechanism worsening prognosis in myocardial infarction patients. Accordingly, dissecting the molecular mechanisms underlying MIRI and formulating effective therapeutic interventions are of great clinical significance. *Lycium barbarum* polysaccharide (LBP), the primary active constituent of *Lycium barbarum*, has garnered considerable attention in the prevention and treatment of cardiovascular diseases due to its anti-inflammatory, antioxidant, vasomotor function-improving, and antithrombotic properties. This study aims to investigate the ability of LBP to alleviate MIRI, with a specific focus on its role in modulating the NOD-like receptor family pyrin domain containing 3 (NLRP3) inflammasome. Myocardial ischemia/reperfusion (I/R) models in rats and hypoxia/reoxygenation (H/R) models in H9c2 cells were established. Myocardial injury and the therapeutic effect of LBP were evaluated by 2,3,5-Triphenyl tetrazolium chloride (TTC) staining, Hematoxylin-eosin (H&E) staining, Terminal deoxynucleotidyl transferase dUTP Nick-End Labeling (TUNEL) staining, and Enzyme-linked immunosorbent assay (ELISA). To elucidate the specific mechanism underlying LBP against MIRI, an Nrf2-overexpressing cell line was generated in H9c2 cells, and pharmacological inhibition of Nrf2 with ML385 was applied for complementary validation. The effects of LBP on H/R-induced oxidative stress, inflammatory response (IL-18, IL-1β), and pyroptosis-related protein expression (NLRP3, apoptosis associated speck-like protein containing a CARD (ASC), cysteine-dependent aspartate-specific proteases (caspase)-1, Gasdermin D (GSDMD)) were systematically evaluated. LBP administration conferred robust cardioprotection in I/R rats, as evidenced by a significant reduction in myocardial infarct size, improved preservation of myocardial fiber architecture, and attenuated leakage of cardiac injury biomarkers (lactate dehydrogenase (LDH) and creatine kinase-MB (CK-MB)). Mirroring these in vivo findings, LBP pretreatment effectively shielded H9c2 cardiomyocytes from H/R insult, markedly enhancing cell viability while curtailing reactive oxygen species (ROS) accumulation and apoptotic activation. A pivotal finding was the pronounced downregulation of Nrf2 in the H/R group, a deficit that was conclusively reversed by LBP treatment. To decisively establish a causal role for Nrf2, we employed a loss-of-function approach; Nrf2 inhibition completely abrogated the protective benefits of LBP, culminating in exacerbated tissue damage, a surge in ROS, and the upregulation of key pyroptosis effectors (NLRP3, ASC, caspase-1, GSDMD). Conversely, a complementary gain-of-function experiment demonstrated that Nrf2 overexpression alone was sufficient to mimic LBP’s effects, significantly blunting H/R-induced ROS production and pyroptosis. LBP alleviates MIRI by inhibiting pyroptosis through activating the Nrf2/NLRP3 axis, thus representing a promising therapeutic candidate for ischemic heart disease with the potential to improve patient outcomes.

## 1. Introduction

MIRI is common in patients with ischemic heart disease (IHD) after revascularization. It manifests as various symptoms including reduced cardiac function, arrhythmias, and ultrastructural changes in myocardial cells [1]. Currently, therapeutic outcomes for MIRI remain suboptimal [2]. Consequently, identifying safe and effective therapeutic agents and elucidating their protective mechanisms holds significant clinical importance for mitigating MIRI.

Natural products offer a distinct therapeutic advantage in cardiovascular diseases through their multi-target and multi-pathway mechanisms, which enable holistic modulation of complex pathological networks while maintaining a favorable safety profile. *Lycium barbarum* polysaccharides (LBP), the primary active component of goji berries, exhibits multiple pharmacological effects [3], including anti-inflammatory and antioxidant properties, improvement in vascular function, and inhibition of thrombogenesis. It holds promising prospects in the field of cardiovascular disease [4,5,6]. It was previously demonstrated that LBP can protect the myocardium from ischemia and hypoxia injury [7]. In recent years, the role of LBP in pyroptosis has garnered widespread attention. LBP can inhibit mitochondrial dysfunction and hepatocyte pyroptosis induced by cadmium [8]. Based on its synergistic inhibition of inflammation and pyroptosis, LBP is proposed as a potential therapeutic candidate for MIRI, though the precise molecular mechanisms underlying its protective effects require further elucidation.

The inflammatory response permeates the entire pathophysiological process of MIRI, and pyroptosis is one of the key mechanisms mediating inflammation in ischemic heart disease. In recent years, pyroptosis has been closely associated with MIRI by researchers [9]. As shown in previous studies, the mechanism of pyroptosis primarily relies on the NLRP3/caspase-1/GSDMD signaling pathway. Knockout of either caspase-1 or NLRP3 reduces early mortality after myocardial infarction in mice, mitigates inflammatory responses, and improves myocardial function [10]. Given the significant role of pyroptosis in MIRI, targeting the pyroptosis signaling pathway is an effective therapeutic strategy. Therefore, LBP might alleviate MIRI by regulating pyroptosis.

Nuclear factor E2-related factor 2 (Nrf2) is recognized as a master regulator of the anti-inflammatory response and plays a pivotal role in the pathogenesis and progression of cardiovascular diseases [11]. Nrf2 knockout rats exhibited significantly increased susceptibility to pyroptosis in the MIRI Model [12]. As found in previous studies, activating Nrf2 alleviates neuroinflammation and renal ischemia–reperfusion injury by inhibiting NLRP3 inflammasome and pyroptosis [13,14]. Thus, whether LBP alleviates MIRI by targeting Nrf2 to suppress the NLRP3-mediated pathway warrants investigation.

We aimed to evaluate the protective effect of LBP against MIRI and investigate whether it achieves this by inhibiting pyroptosis. We demonstrated that LBP significantly upregulates Nrf2 expression and attenuates NLRP3 inflammasome activation. Mechanistically, the Nrf2/NLRP3 signalling pathway is the key mediating mechanism for LBP-induced pyroptosis inhibition.

## 2. Results

### 2.1. The Alleviation of Myocardial Injury by LBP in MIRI Rat Model

**Figure 1:**
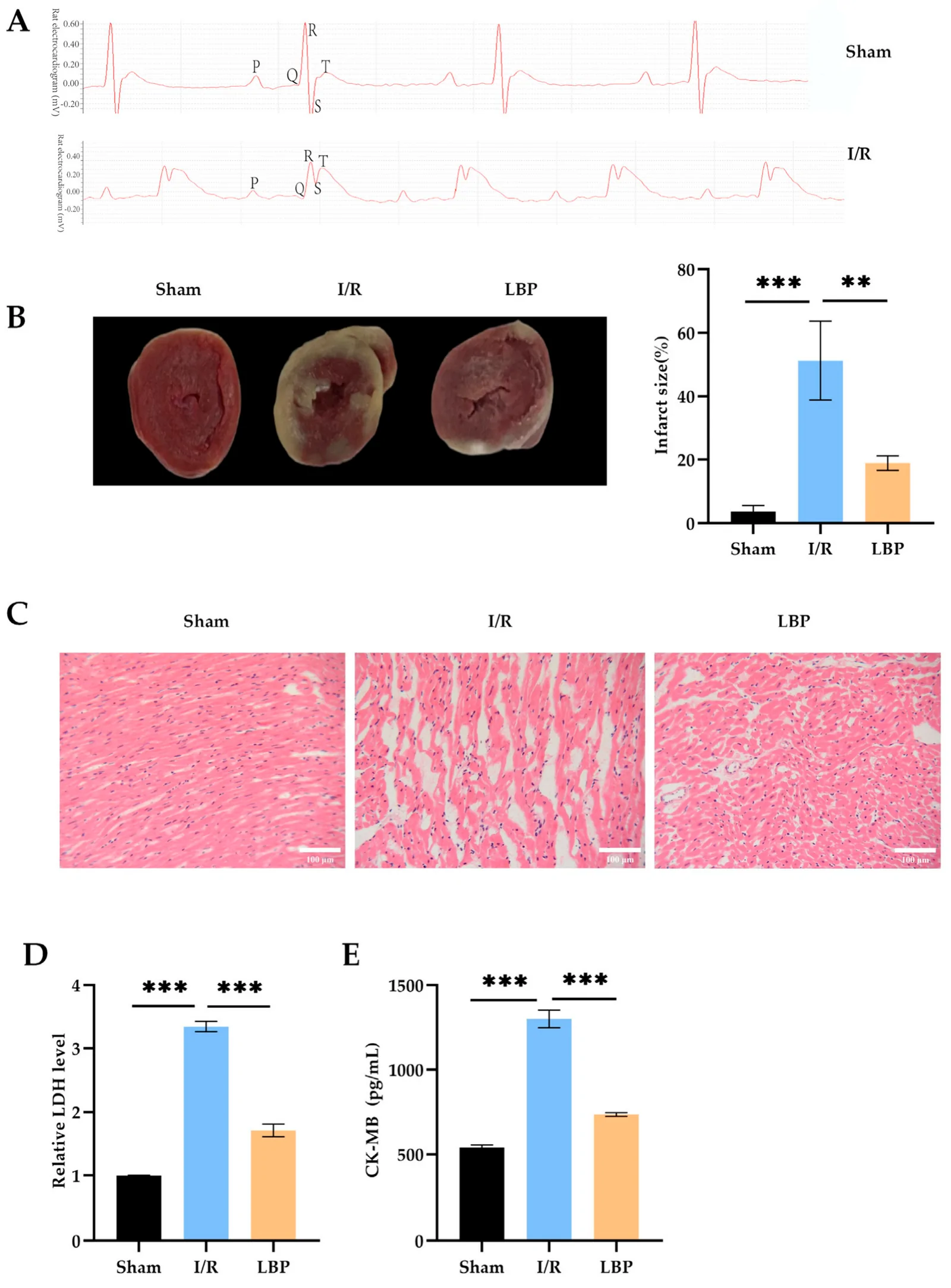
The alleviation of myocardial injury by LBP in MIRI rat model. (**A**) ECG of rats. (**B**) TTC staining. (**C**) H&E staining; scale bar = 100 μm. (**D**) LDH levels. (**E**) CK-MB levels. Data are shown as mean ± SD (*n* = 6). ** *p* < 0.01; *** *p* < 0.001.

Myocardial I/R models were established by ligating the left anterior descending coronary artery for 30 min followed by reperfusion for 2 h. ECG analysis of rats revealed significant ST-segment elevation in the I/R group, compared to the sham group (Figure 1A), demonstrating successful establishment of the MIRI rat model. TTC staining was performed to evaluate the impact of LBP on myocardial infarction area in MIRI rats. Results showed that a significant increase in myocardial infarction area was observed in the I/R group compared to the sham group. The LBP group exhibited a significantly reduced myocardial infarction area compared to the I/R group (Figure 1B). Myocardial fiber morphology was evaluated to investigate the effect on myocardial fibers and inflammation via H&E staining. As shown in Figure 1C, rats in the sham group exhibited regular arrangement of myocardial fibers, clearly defined cytoplasm and nucleus. In contrast, broken and disorganized myocardial fibers were observed in the I/R group, accompanied by significant neutrophil infiltration and capillary congestion between myocardial fibers. In contrast to the I/R group, characterized by severe myocardial damage, the LBP group displayed significantly preserved tissue architecture with no evidence of inflammatory cell infiltration (Figure 1C). In addition, we quantified serum levels of myocardial injury markers (LDH and CK-MB). The levels of both markers were significantly higher in the I/R group than in the sham group. However, this increase was markedly attenuated following LBP administration (Figure 1D,E). The findings indicated that LBP treatment significantly ameliorated myocardial injury induced by MIRI in rats.

### 2.2. LBP Inhibits NLRP3 Inflammasome Activation to Alleviate Pyroptosis

**Figure 2:**
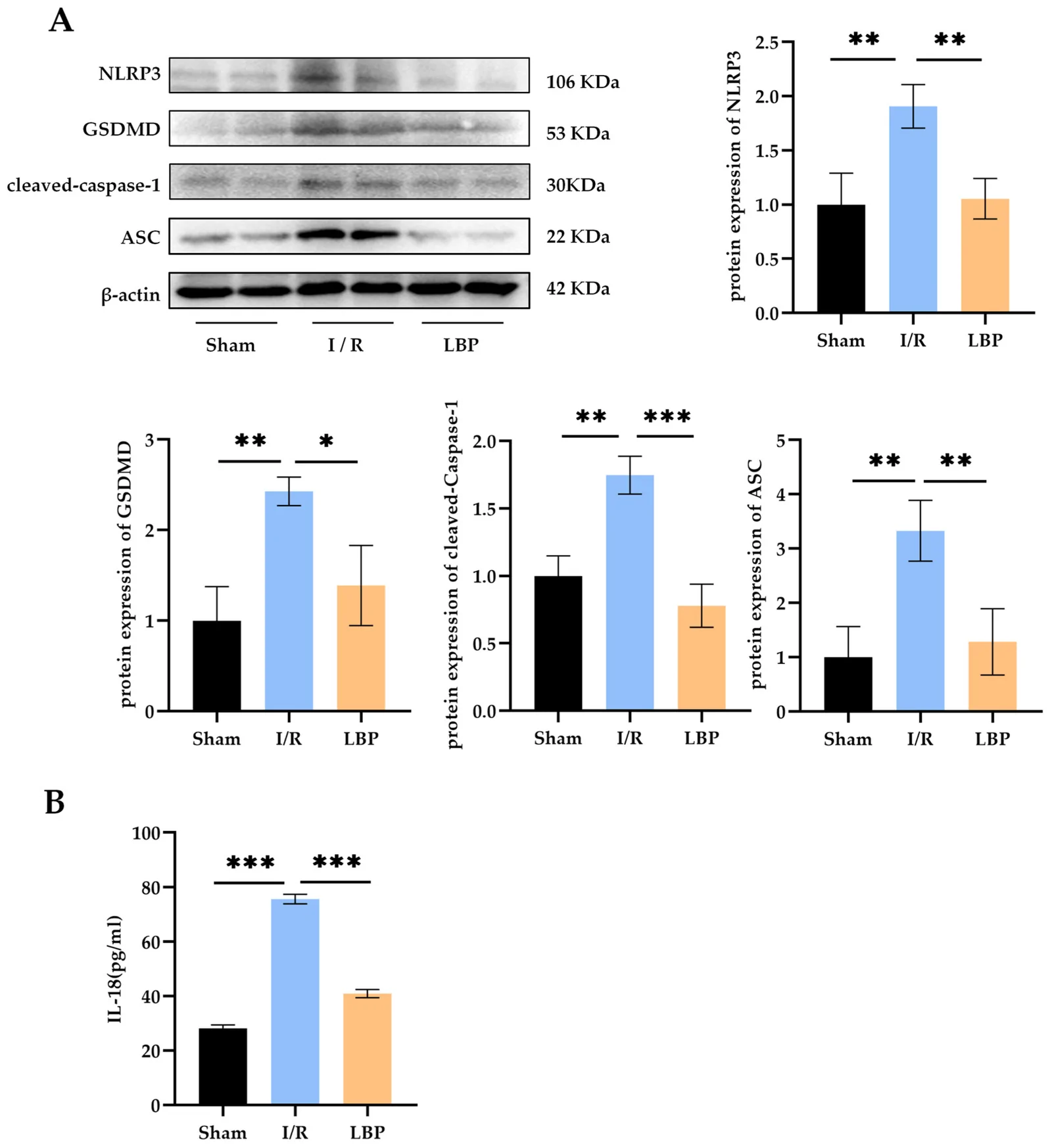
Effects of LBP on pyroptosis induced by I/R in rats. (**A**) Protein expression levels of NLRP3, GSDMD, cleaved-caspase-1, ASC. (**B**) Serum IL-18 levels. Data are shown as mean ± SD (*n* = 6). * *p* < 0.05; ** *p* < 0.01; *** *p* < 0.001.

Pyroptosis represents one of the principal forms of regulated cell death implicated in cardiac I/R injury [15]. The NLRP3 inflammasome, a multiprotein complex assembled by NLRP3 protein, adaptor protein ASC and effector protein pro-caspase-1, is a key upstream switch that triggers pyroptosis. In terms of therapeutic strategies, targeted inhibition of the assembly or activity of NLRP3 inflammasome is considered as a new direction with great potential to alleviate MIRI. Hence, we evaluated the effect of LBP on NLRP3 inflammasome. The protein levels of NLRP3, cleaved-caspase-1, and ASC were markedly upregulated in rat cardiac tissue following I/R injury, accompanied by a marked increase in the levels of GSDMD, a downstream protein and key factor in pyroptosis. As shown in Figure 2A, compared with the Sham group, the expression levels of NLRP3, GSDMD, Caspase-1 and ASC in cardiomyocytes were significantly increased after I/R injury. I/R injury significantly induced pyroptosis. However, these changes in pyroptosis-associated protein levels were reversed in rats pretreated with LBP. Activated GSDMD forms pores in the plasma membrane, leading to osmotic swelling and rupture of the cell, thereby releasing the highly pro-inflammatory cytokine IL-18 into the extracellular. As shown in Figure 2B, the serum IL-18 level was abnormally increased in rats undergoing I/R injury, while it was significantly decreased in rats pretreated with LBP. The results indicated that LBP significantly ameliorates pyroptosis induced by I/R injury, with this protective effect mediated through the inhibition of NLRP3 inflammasome activation.

### 2.3. LBP Alleviates Pyroptosis Induced by H/R Injury

**Figure 3:**
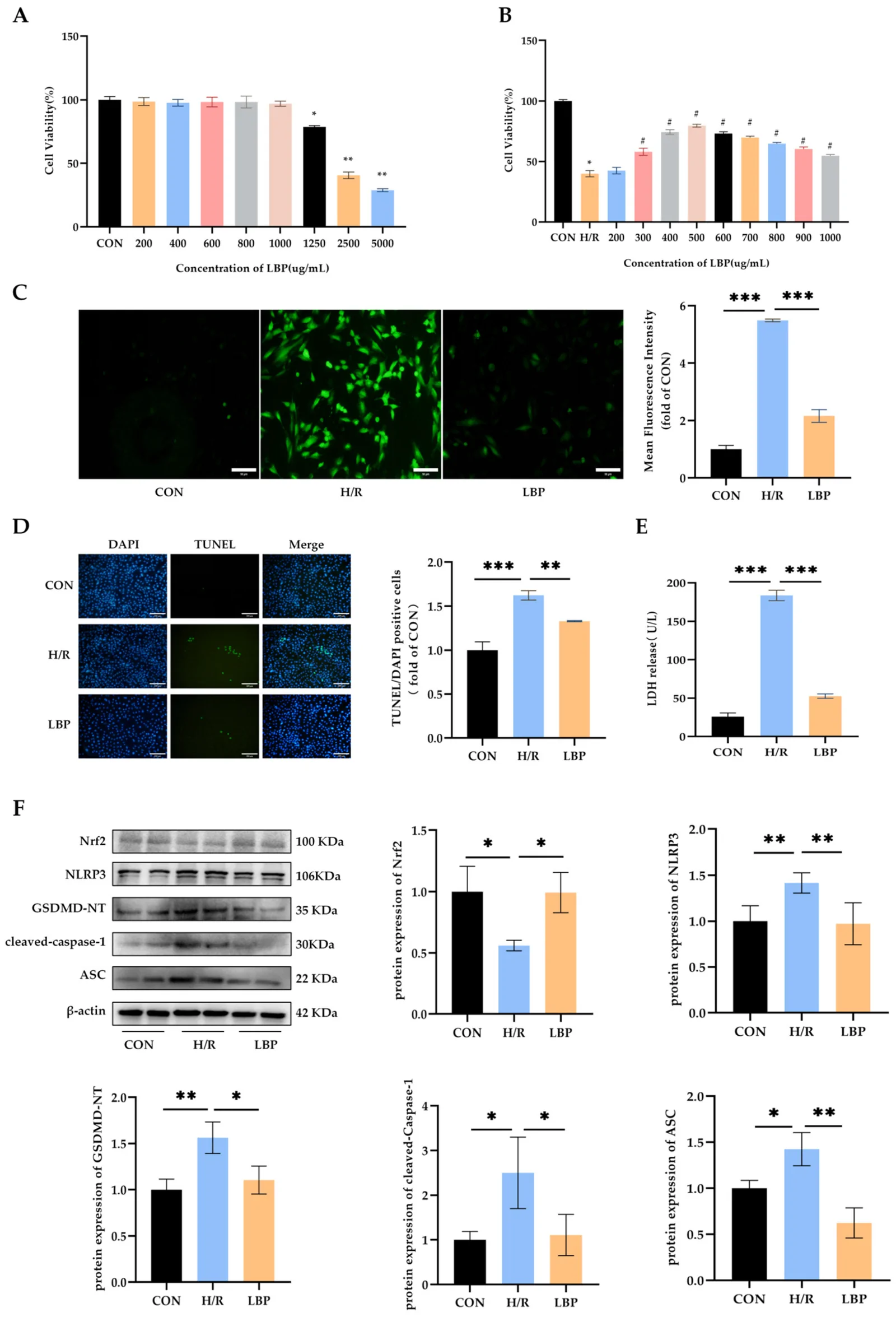
Effects of LBP on H/R injury in H9c2 cells. (**A**,**B**) CCK-8 assay; (**C**) ROS assay; scale bar = 50 μm. (**D**) TUNEL staining; scale bar = 200 μm. (**E**) LDH levels. (**F**) Protein expression levels of Nrf2, NLRP3, GSDMD, cleaved-caspase-1, ASC. Data are shown as mean ± SD (*n* = 6). vs. CON * *p* < 0.05; ** *p* < 0.01; *** *p* < 0.001; vs. H/R ^#^ *p* < 0.05.

Hypoxia/reoxygenation model was used to simulate MIRI injury in vitro. H9c2 cells were treated with different concentrations of LBP, and the cell viability was detected by CCK8 method to screen the optimal concentration of LBP. The findings revealed that there were no significant changes in cell viability in the LBP concentration range of 0 to 1000 μg/mL (Figure 3A). The results demonstrated that LBP has a good safety profile. H/R intervention was performed 24 h after treatment with different concentrations of LBP. Results showed that the highest cell viability was achieved with 500 μg/mL LBP treatment (Figure 3B). Therefore, we used 500 μg/mL as the concentration for subsequent administration.

To explore the role of LBP in H/R injury, we examined its effects on apoptosis by measuring ROS production. ROS levels were markedly elevated in cardiomyocytes following H/R injury relative to the CON group. In contrast, the ROS level was lower in LBP group than in those subjected to H/R injury (Figure 3C). H/R injury markedly increased the proportion of TUNEL-positive cells, which was significantly reversed by LBP treatment (Figure 3D). ELISA assay revealed that LBP treatment significantly attenuated the LDH leakage induced by H/R injury, thereby alleviating cardiomyocyte damage (Figure 3E). Consistent with the in vivo findings, in vitro assays demonstrated that LBP attenuates MIRI by suppressing cardiomyocyte apoptosis.

Nrf2 is an important negative regulator of NLRP3. Therefore, we examined not only the expression of pyroptosis-related proteins (NLRP3, caspase-1, ASC, and GSDMD) but also that of the key antioxidant protein Nrf2. At the molecular level, H/R injury significantly increased the levels of pyroptosis-associated proteins but decreased Nrf2 expression. LBP concurrently reversed the increase in pyroptosis-related proteins and up-regulated Nrf2 protein expression, mediated through enhanced cellular antioxidant activity (Figure 3F). We conclude that LBP protects from MIRI injury by attenuating pyroptosis, an underlying mechanism that involves suppression of the NLRP3 inflammasome pathway.

### 2.4. Effect of LBP on the Nrf2/NLRP3 Signaling Pathway in H9c2 Cells

**Figure 4:**
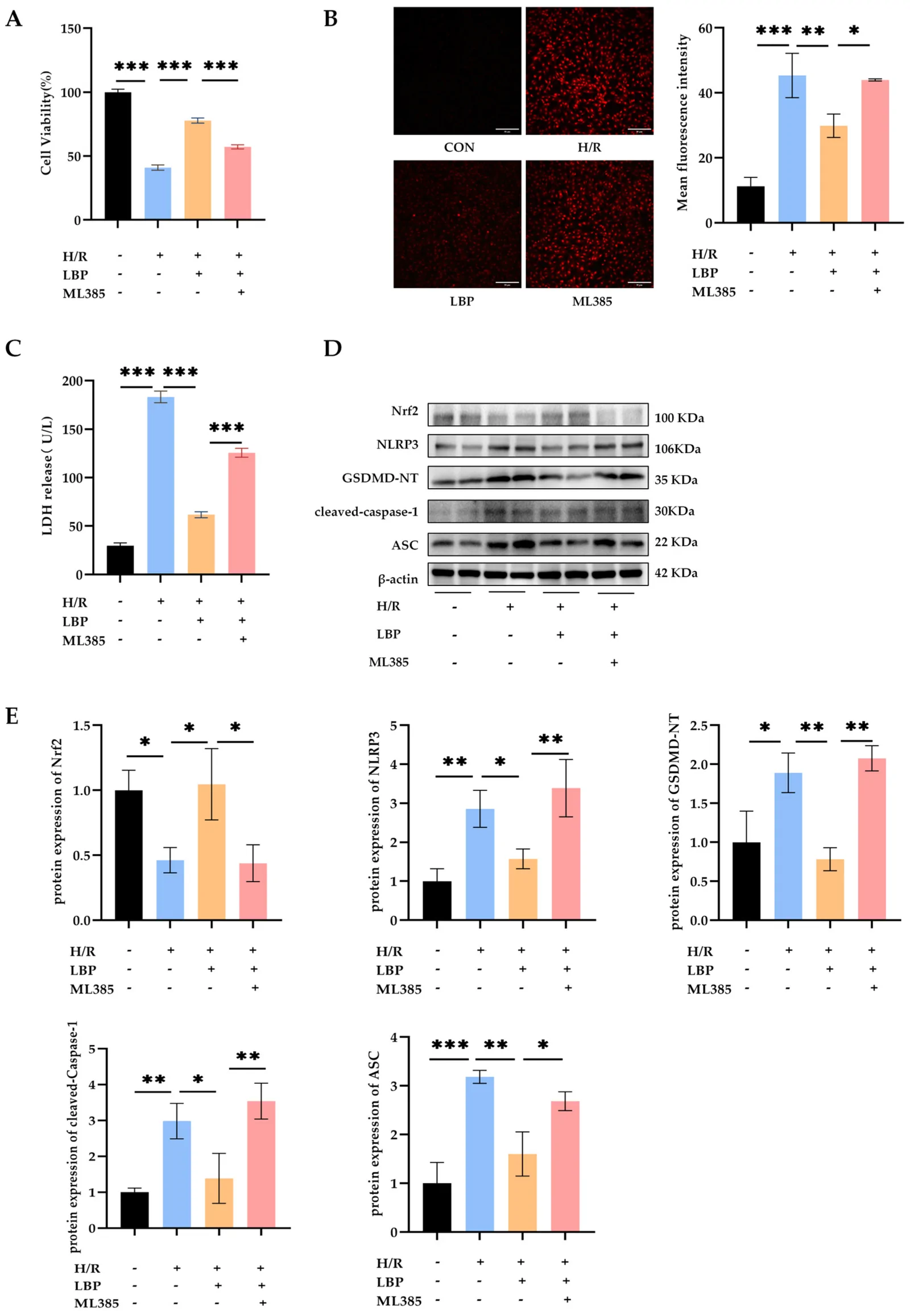
Effect of Nrf2 inhibition on LBP alleviating H/R injury in H9c2 cells. (**A**) Cell viability. (**B**) ROS assay; scale bar = 50 μm. (**C**) LDH levels. (**D**,**E**) Protein expression levels of Nrf2, NLRP3, GSDMD, cleaved-caspase-1, ASC. Data are shown as mean ± SD (*n* = 6). * *p* < 0.05; ** *p* < 0.01; *** *p* < 0.001.

To further elucidate the mechanism underlying the protective effect of LBP against MIRI, ML385, an Nrf2 inhibitor, was used to pretreat H9c2 cells. The cell viability results demonstrated that ML385 reduced the viability of cardiomyocytes, indicating that the protective effect of LBP against H/R injury was blocked by ML385 (Figure 4A). Assessment of intracellular ROS showed that the effect of LBP on ROS levels was significantly counteracted by ML385 (Figure 4B). ML385 also increased LDH release, further confirmed that LBP’s protective effect was inhibited by ML385 (Figure 4C). To further elucidate the mechanism by the protection of LBP to MIRI, ML385, an Nrf2 inhibitor, was used to pretreat H9c2 cells. Nrf2 upregulation induced by LBP was effectively reversed by ML385 (Figure 4D,E). The expression levels of pyroptosis-associated proteins were further validated. Compared with the LBP group, NLRP3, caspase-1, ASC, and GSDMD, the pyroptosis-related proteins were significantly elevated in the ML385 intervention group (Figure 4D,E), indicating that the suppression of pyroptosis by LBP was reversed by inhibiting Nrf2. This study demonstrated that LBP inhibited pyroptosis via Nrf2 pathway activation, thereby alleviating myocardial injury.

### 2.5. Effect of LBP on the Nrf2/NLRP3 Signaling Pathway in Rats

**Figure 5:**
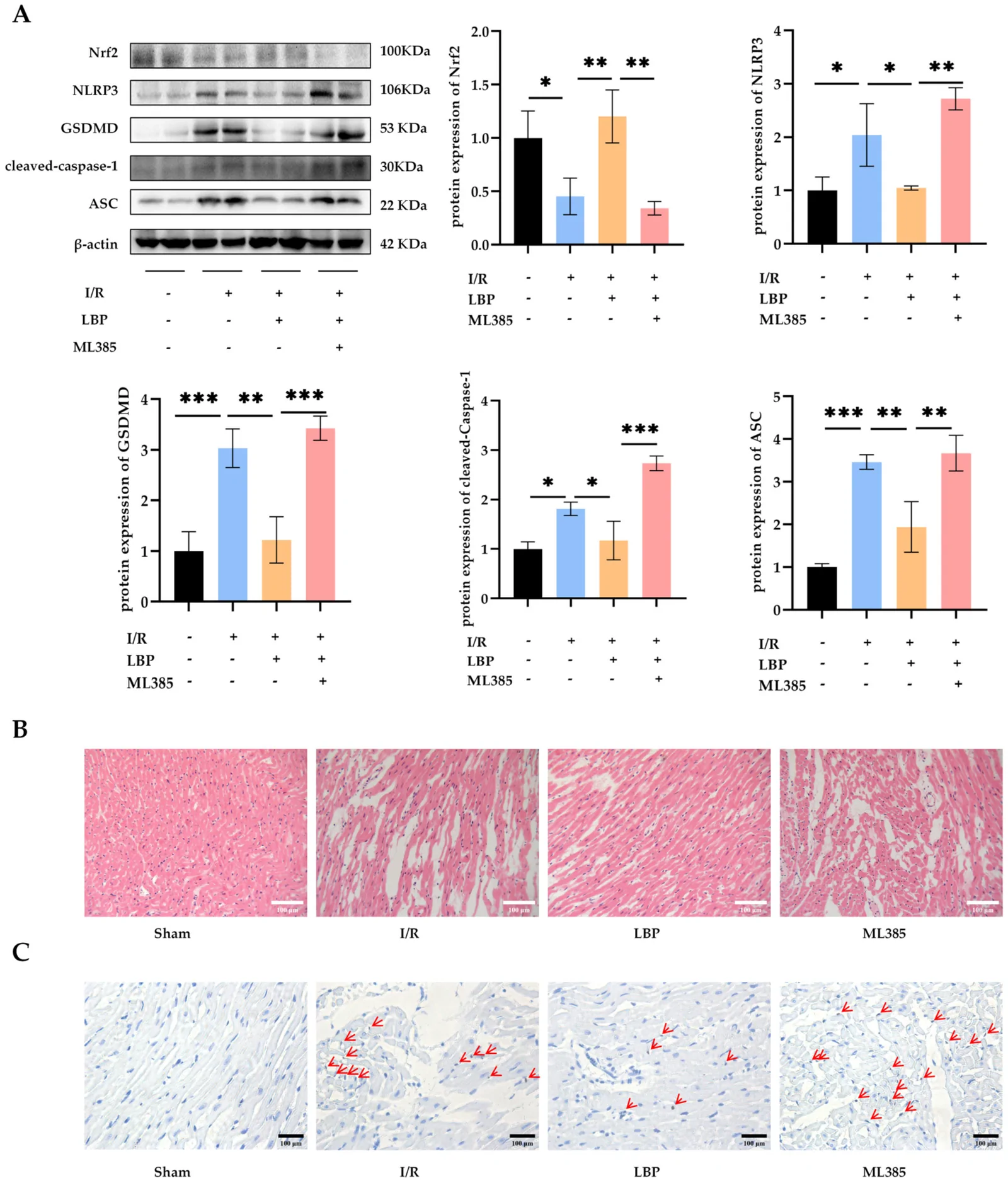
Effect of Nrf2 inhibition on LBP alleviating MIRI in rats. (**A**) Protein expression levels of Nrf2, NLRP3, GSDMD, cleaved-caspase-1, ASC. (**B**) H&E staining; scale bar = 100 μm. (**C**) TUNEL staining; scale bar = 100 μm. Data are shown as mean ± SD (*n* = 6). * *p* < 0.05; ** *p* < 0.01; *** *p* < 0.001.

The conclusions drawn from the mechanism verification in vitro are also verified in vivo. Consequently, co-treatment with ML385 reversed LBP-mediated suppression of the NLRP3 inflammasome pathway (NLRP3, ASC, caspase-1, GSDMD) and reversed the upregulation of Nrf2 (Figure 5A). H&E staining revealed extensive cardiomyocyte rupture and disarray, as well as increased neutrophil infiltration and inflammation, in the ML385 intervention group (Figure 5B). ML385 increased apoptosis compared to the LBP group, as demonstrated by TUNEL results (Figure 5C). Consistent with the in vitro results, this finds further confirmed that LBP ameliorated myocardial injury by activating the Nrf2 pathway, thereby inhibiting pyroptosis.

### 2.6. LBP Alleviates NLRP3 Dependent Pyroptosis by Activating Nrf2

**Figure 6:**
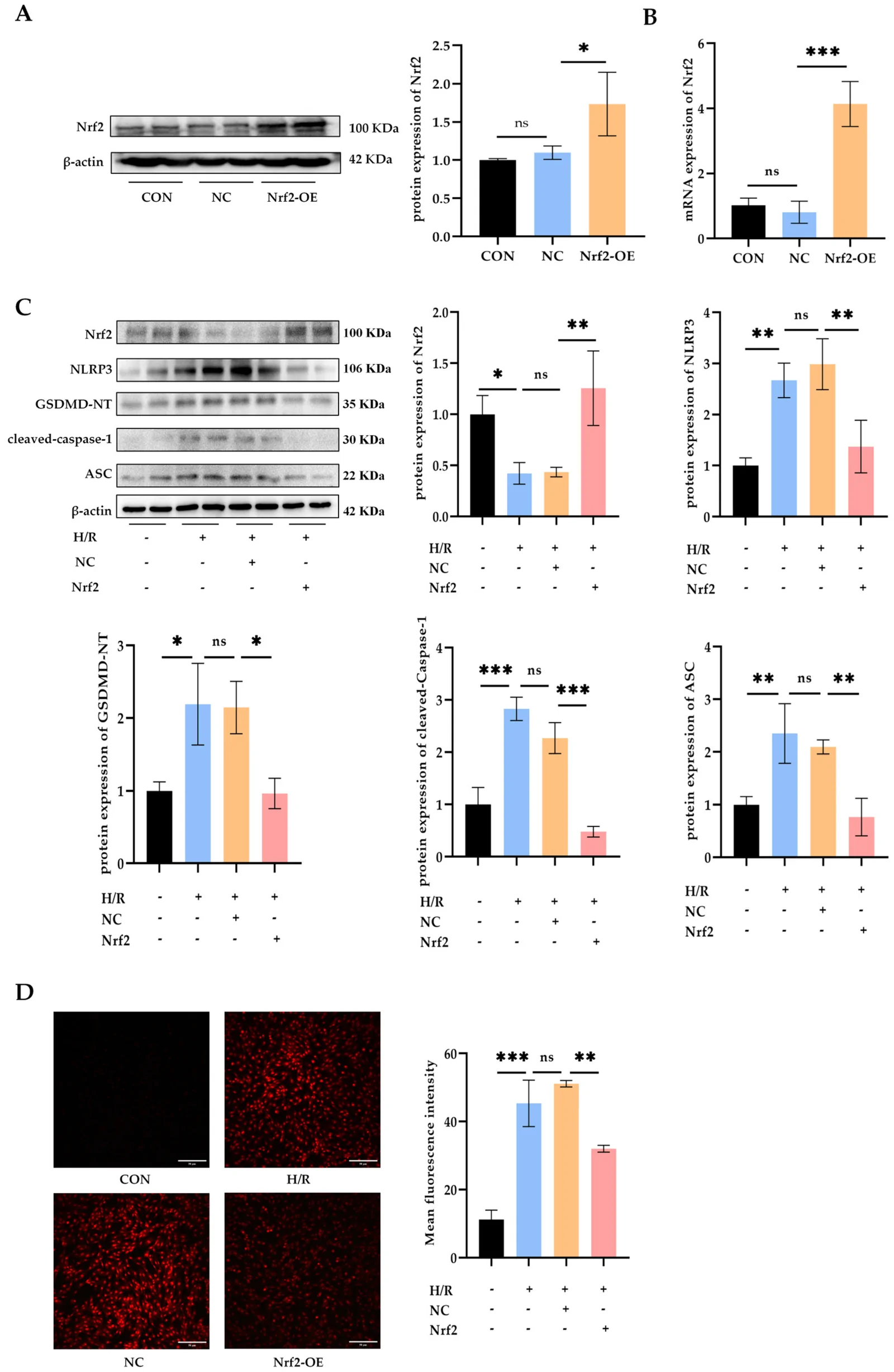
Effect of Nrf2 activation on pyroptosis induced by H/R in H9c2 cells. (**A**) mRNA expression of Nrf2. (**B**) Protein expression of Nrf2. (**C**) Protein expression levels of Nrf2, NLRP3, GSDMD, cleaved-caspase-1, ASC. (**D**) ROS assay; scale bar = 50 μm. Data are shown as mean ± SD (*n* = 6). * *p* < 0.05; ^**^ *p* < 0.01; *** *p* < 0.001; ns means no statistical difference.

To elucidate the mechanistic role of Nrf2 in MIRI, a stable Nrf2-OE H9c2 cell line was established via lentiviral transduction. We successfully established a stable Nrf2-OE H9c2 cell line, as evidenced by significantly enhanced Nrf2 expression at both the mRNA and protein levels (Figure 6A,B). We examined the expression of proteins related to pyroptosis. Results showed that Nrf2-OE significantly suppressed the expression of pyroptosis-related proteins (Figure 6C). Furthermore, a pronounced decrease in intracellular ROS was detected upon Nrf2-OE (Figure 6D). In conclusion, the results demonstrated that LBP treatment mitigated cardiomyocyte injury induced by H/R by activating Nrf2 to inhibit NLRP3-dependent pyroptosis.

## 3. Discussion

In addition to classical antioxidant strategies such as spin traps, SOD, and catalase that have been extensively studied since the 1980s and 1990s for mitigating reperfusion injury [16,17,18], naturally derived plant extracts have gained increasing attention for their multi-target cardioprotective effects. Natural compounds possess excellent antioxidant and anti-inflammatory properties and are considered to have potential for improving cardiovascular diseases [19,20]. LBP, the principal bioactive constituent of Goji berry, exhibits pharmacological effects including antioxidant, antibacterial, anti-inflammatory, immunomodulatory, and neuroprotective activities, offering broad application prospects in both food and medical fields [21,22]. Previous studies indicated that LBP alleviated ischemia–reperfusion injury in multiple rat organs by counteracting oxidative stress and modulating inflammatory signaling pathways [23,24], indicating the critical role of LBP pretreatment in ischemic injury. Our findings from both in vitro and in vivo experiments revealed that LBP possesses a protective effect against ischemia/reperfusion injury.

**Figure 7:**
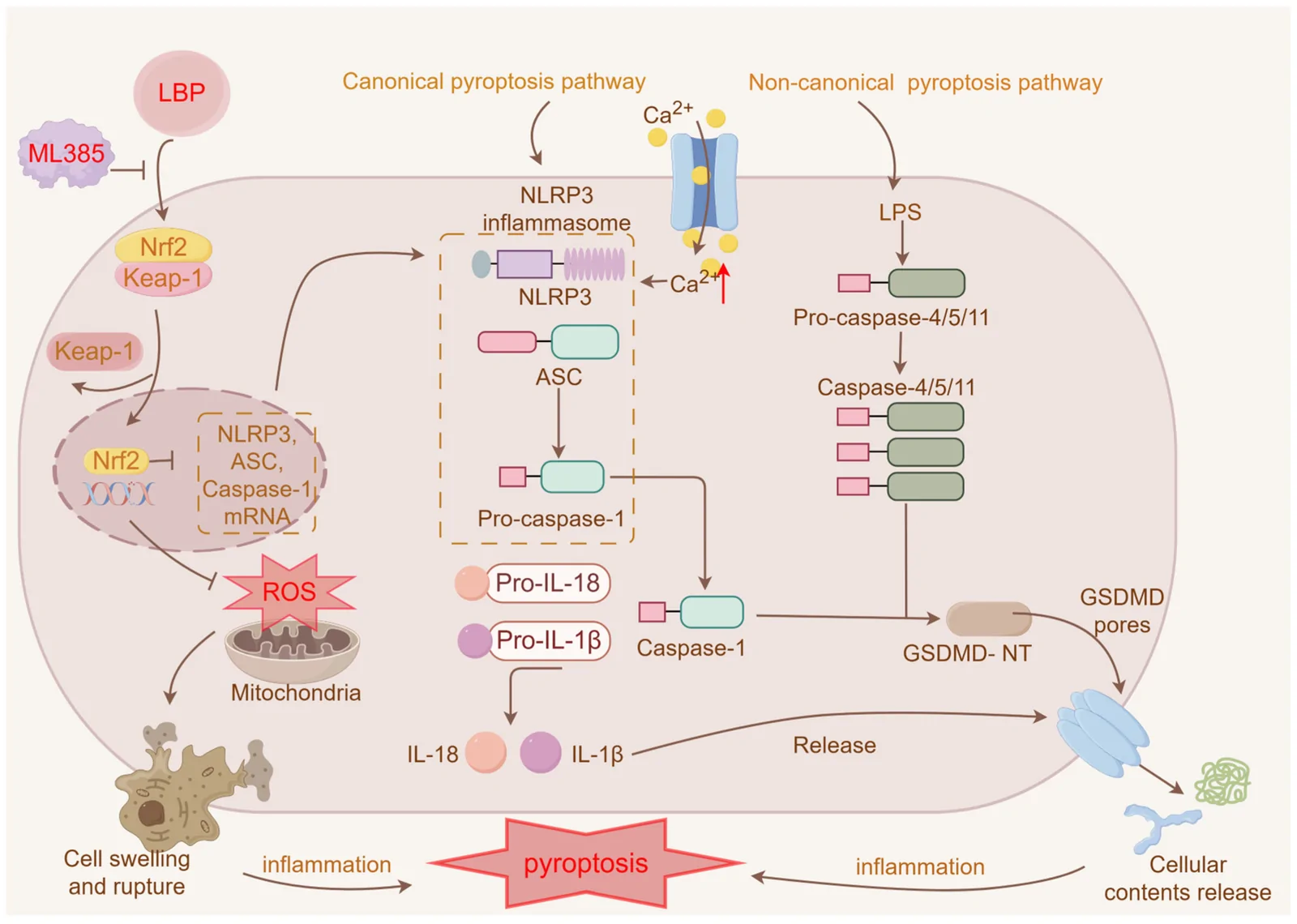
Molecular mechanism of LBP in treatment for pyroptosis in MIRI. Supported and produced by Figdraw.

Pyroptosis, serving a critical function in immune defense as well as disease prevention, is a current focus of biomedical research. The specific mechanism relies on two core pathways: canonical and non-canonical pyroptosis pathways. Both of these pathways ultimately activate GSDMD, the executioner protein of pyroptosis. Figure 7 clearly illustrates the two core signaling pathways and their key steps. Increasing research has revealed the detrimental role of pyroptosis triggered by NLRP3 inflammasome activation in MIRI. Inhibiting pyroptosis, such as by targeting the NLRP3 inflammasome or GSDMD, is considered a novel therapeutic strategy for MIRI [16]. We hypothesized that a cardioprotective effect of LBP in MIRI may be mediated by inhibiting pyroptosis through suppression of NLRP3 inflammasome activation. We found that, LBP markedly downregulated the protein levels of the NLRP3 inflammasome (NLRP3, ASC, caspase-1) and its pyroptotic effector (GSDMD) in MIRI. GSDMD, the pyroptosis executioner protein, can oligomerize on the cell membrane to form pores after cleavage by activated caspase-1. These GSDMD pores cause an ion imbalance (Na^+^, Ca^2+^ influx), allowing massive water influx that ultimately ruptures the cell membrane. Consequently, mature IL-1β, IL-18, and other intracellular proinflammatory mediators are released into the extracellular space [25,26]. We thus examined the levels of IL-1β and IL-18. We found that the release of these mediators was inhibited significantly by LBP. In conclusion, we demonstrated that LBP has therapeutic potential for cardiac protection by inhibiting pyroptosis, which provides a theoretical basis for its application in treating MIRI.

Nrf2 is a well-established upstream negative regulator of NLRP3 [27]. Accumulating evidence indicates that Nrf2 suppresses NLRP3-mediated inflammatory signaling through both antioxidant mechanisms and direct transcriptional regulation, thereby mediating cellular responses to pathophysiology [28,29]. The Nrf2/NLRP3 regulatory axis has been extensively characterized in cardiovascular disease models [29,30]. Notably, We employed a combined strategy involving Nrf2 inhibition (ML385) and Nrf2 overexpression (Nrf2-OE). Using the complementary approaches of gain-of-function and loss-of-function assays, we clearly demonstrated that LBP exerts its cardioprotective effects by activating Nrf2 and subsequently inhibiting NLRP3-mediated pyroptosis.

We demonstrated that it is through Nrf2 activation that LBP alleviates NLRP3-dependent pyroptosis, which was validated by Nrf2 inhibition and overexpression experiments. However, MIRI is a complex pathological process involving the interaction of multiple signaling pathways. Although displaying multi-target and multi-pathway effects, the hierarchical relationships among LBP’s distinct mechanisms and the network-based regulatory patterns governing them remain incompletely elucidated. Additionally, a comprehensive approach is often employed in the clinical treatment of MIRI. Whether the combination of LBP with traditional Western medicines (including antioxidants and ion channel modulators) or other active components of traditional Chinese medicine (including ginsenosides and berberine) produces synergistic or antagonistic effects remains to be systematically investigated. In the future, it is necessary to further clarify the mechanism of action of LBP and its drug combination effects, which will provide more robust experimental evidence for its cardiovascular therapeutic applications.

## 4. Materials and Methods

### 4.1. Reagents

LBP was commercially obtained from Shanghai Yuanye Bio-Technology Co., Ltd. (s31394, Shanghai, China). H9c2 cardiomyocytes were kindly provided by the research group of Professor Guangyuan Yang at the Jiamusi University (Jiamusi, China). The ELISA kits for detecting CK-MB (SEA479Ra), IL-1β (SEA563Ra), and IL-18 (SEA064Ra) were procured from Uscn Life Science Inc. (Wuhan, China). Nrf2 overexpression (Nrf2-OE) lentivirus, control lentivirus, and polybrene were provided by Genomeditech Co., Ltd. (Shanghai, China). ML385 was supplied by Proteintech Group, Inc. (Wuhan, China). We purchased the TUNEL kit, DCFH-DA probe, LDH kit, CCK8 solution, TTC staining solution, and hematoxylin and eosin (H&E) staining solution from Beyotime Biotechnology Co., Ltd. (Shanghai, China). We purchased dihydroethidium (DHE) from MedChemExpress (Monmouth, NJ, USA). Anti-Nrf2 (bs1074R, 1:500), anti-ASC (bs41334R, 1:500), and anti-GSDMD (bs-14287R, 1:500) antibodies were provided by Beijing Bio-Ocean Biotechnology Co., Ltd. (Beijing, China). We purchased anti-NLRP3 (WL02635, 1:1000), GSDMD-NT (WL05411, 1:1000) and Caspase-1 (WL02996, 1:1000) antibodies from Wanleibio Co., Ltd. (Shenyang, China). And we got antibody of anti-β-actin (66009-1-Ig, 1:50,000) from Proteintech Group, Inc. (Wuhan, China). Antibody of Goat anti-rabbit IgG, peroxidase conjugated (NC-AP132P, 1:10,000), was provided by Nachem Biotechnology Co., Ltd. (Harbin, China). Horseradish enzyme-labeled goat anti-mouse IgG (H + L) (A0216, 1:1000) was obtained from Beyotime Biotechnology Co., Ltd. (Shanghai, China). Methanol and anhydrous ethanol were supplied by Fuyug Chemicals Co., Ltd. (Tianjin, China).

### 4.2. Animals

We obtained male Sprague Dawley (SD) rats (220 ± 30 g) from Changchun Yisi Experimental Animal Technology Co., Ltd. (Changchun, China). The animals were raised in the Animal Experiment Center of Jiamusi University under specific pathogen-free (SPF) conditions and a 12-h light–dark cycle. All experimental animals underwent a 7-day acclimatization period prior to subsequent experiments. All experimental procedures involving animals were conducted following the guidelines in the Guide for the Care and Use of Laboratory Animals and received official approval from the Animal Experimental Ethics Committee of Jiamusi University (Approval No. JDJCYXY2023029).

### 4.3. Myocardial Ischemia/Reperfusion Model

Rats were anesthetized with 1.5% sodium pentobarbital (30 mg/kg). Subsequently, the rats were attached to a miniaturized breathing machine. (Chengdu Taimeng Software Co., Ltd., Chengdu, China). Continuous electrocardiogram (ECG) signals of cardiac electrical activity were recorded using needle electrodes placed subcutaneously in the limbs. After opening the thoracic cavity, 6-0 surgical suture was used to ligate the left anterior descending coronary artery for 30 min to achieve myocardial ischemia, followed by release of the ligature for 2 h of reperfusion. Sham surgery served as the control group, involving only suturing without ligation, while all other procedures were identical to those in the model group. The criteria for establishing a successful MIRI model were as follows: during ischemia, myocardial tissue appeared pale and cyanotic, and the ST segment on ECG is significantly elevated. With reperfusion, the ischemic myocardial tissue turned red, and the ST segment returned to normal.

### 4.4. Animal Groups

**Figure 8:**
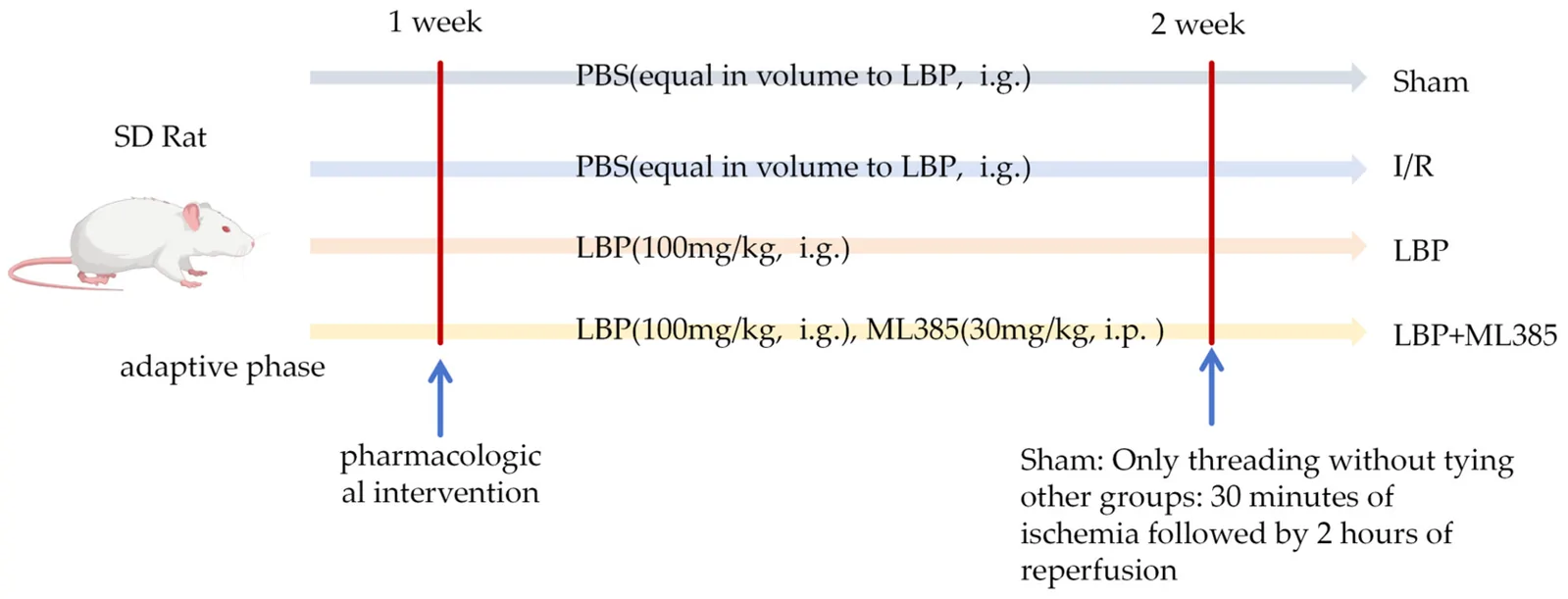
SD rat drug intervention diagram.

Rats were randomly assigned to 4 groups. Rats in the sham-operated (Sham) group only underwent suture placement under the left anterior descending coronary artery without ligation. Rats in the model (I/R) group were subjected to 30 minutes of left anterior descending coronary artery ligation, followed by 2 hours of reperfusion. Rats in the LBP (LBP) group were intragastrically administered LBP (100 mg/kg/day) for 7 consecutive days [24], followed by the establishment of the I/R model. Rats in the Nrf2 inhibition (ML385) group were first intraperitoneally injected with ML385 (30 mg/kg), And 2 h later, LBP was administered by intragastric gavage. Both drugs were given continuously for 7 days, after which the I/R model was established. All groups except the LBP group and the ML385 group were administered an equal volume of PBS intragastrically for 7 consecutive days. LBP was dissolved in PBS before use. The animal experimental protocol is illustrated in Figure 8.

### 4.5. TTC Staining

After the procedure, the rats were euthanised and their hearts were removed. Hearts were sectioned into 5–6 transverse slices of equal thickness (approximately 2 mm). Following incubation in 1% TTC in the dark (37 °C, 20 min), the slices were fixed for analysis. The slices were washed twice with PBS. Infarcted myocardium appears white, while viable tissue appears red. The percentage of infarct area was analyzed using ImageJ software 1.54 (National Institutes of Health, NIH, Bethesda, MD, USA).

### 4.6. H&E Staining

After being soaked for 24 h in the 4% paraformaldehyde solution, and then cardiac tissues were embedded in paraffin and sectioned at 5 μm. Sections were then stained with hematoxylin and eosin using a standard protocol. A microscope (Leica, Wetzlar, Germany) was used to capture the H&E-stained images.

### 4.7. Cell Culture

H9c2 cells were maintained under standard culture conditions (37 °C, 5% CO_2_). The culture medium consisted of high-glucose DMEM (Thermo Fisher Scientific, Waltham, MA, USA) enriched with 10% fetal bovine serum (Thermo Fisher Scientific, Waltham, MA, USA) and 1% penicillin–streptomycin (Beyotime, Shanghai, China). Cells were passaged or plated when they are in good condition and have reached 80% confluence.

### 4.8. Lentiviral Transfection

Perform transfection using the multiplicity of infection (MOI) recommended by the reagent manufacturer (MOI = 80). We seeded cells in 24-well plates. Then we infected H9c2 cells with lentivirus carrying Nrf2-OE and the corresponding control virus (NC), respectively. After 24 h, the medium was replaced with fresh complete medium, and the cells were cultured for an additional 72 h. Further culture was maintained with 0.5 μg/mL puromycin dihydrochloride (Beyotime, Shanghai, China).

### 4.9. Cell H/R Model and Selection of Optimal Drug Concentration

#### 4.9.1. Cell H/R Model

H9c2 cells were seeded in 6-well plates. Cells were treated for 3 h in anaerobic conditions using sugar-free DMEM (Thermo Fisher Scientific, Waltham, MA, USA). Then replace with complete medium. Reoxygenation at 0, 1.5, 3, 6, and 9 h, respectively (37 °C, 5% CO_2_). Cell morphological changes were observed at different reoxygenation durations after hypoxia. Since the cell viability was approximately 50% at 3 h of reoxygenation, we selected 3 h of reoxygenation as the modeling condition for subsequent experiments.

#### 4.9.2. Selection of Optimal Drug Concentration

Dissolve 50 mg of Lycium polysaccharide in 10 mL of complete medium to prepare a stock solution of LBP at a concentration of 5000 mg/mL. Store at 4 °C. Seed H9c2 cells into a 96-well plate. After 24 h of incubation in LBP at concentrations of 200, 400, 600, 800, 1000, 1250, 2500, and 5000 μg/mL, CCK-8 working solution was added. Cell viability was measured after 1 h. After determining the safe concentration range of LBP in H9c2 cells, the H/R induced H9c2 cell injury model was further employed to screen and identify the optimal concentration of LBP for its protective effects, which was used in subsequent cell experiments.

### 4.10. Cell Viability Assay

Cells were plated in 96-well plates. After the H/R model was established, 100 μL/well of CCK-8 working solution was added according to the manufacturer’s protocol. Cell viability was measured after 1 h.

### 4.11. Assessment of Cardiomyocyte Injury

The levels of relevant indicators in the samples were determined using kits for enzyme activity detection and ELISA, respectively. The activities of CK-MB and LDH were quantified using commercial assay kits according to the manufacturers’ protocols. Absorbance was measured with a microplate reader (BioTek Instruments, Winooski, WT, USA). The levels of cytokines IL-18 and IL-1β were detected using corresponding ELISA kits.

### 4.12. TUNEL Staining

Cells: H9c2 cells were seeded in 24-well plates and subjected to the respective drug treatments and modeling procedures. Following the interventions, the cells were washed once with PBS and fixed with 4% formaldehyde for 20 min. Then the cells were permeabilized with 0.1% Triton X-100 (Solarbio, Beijing, China) (37 °C, 10 min) and washed again. Subsequently, apoptosis was detected by incubating the cells with 100 μL TUNEL reaction mixture (37 °C, 1 h). Cell nuclei were counterstained with 200 μL DAPI solution (25 °C, 5 min). Finally, the samples were mounted with an anti-fade sealing agent and visualized under a fluorescence microscope (Leica, Wetzlar, Germany).

Tissue: Heart tissues, in 4% formaldehyde solution (Lanjieke Technology Co., Ltd., Suzhou, China), were fixed for 24 h. Subsequently, paraffin embedding and tissue sectioning (5 μm) were performed. Paraffin sections of cardiac tissue were deparaffinized, followed by processing according to the TUNEL apoptosis detection kit protocol. Results were observed under a microscope (Leica, Wetzlar, Germany).

### 4.13. ROS Assay

H9c2 cells were seeded in 6-well plates. Cells were loaded with 1 mL of DCFH-DA or DHE working solution (37 °C, 20–30 min). Finally, fluorescence was visualized under a microscope (Olympus Corporation, Tokyo, Japan).

### 4.14. Western Blotting Analysis

Protein extracts from cardiac tissue and H9c2 cells, obtained using RIPA buffer (Beyotime, Shanghai, China), were quantified and subjected to SDS-PAGE. The separated proteins were transferred to PVDF membranes (Merck KGaA, Darmstadt, Germany), which were then blocked with 5% non-fat milk (Beyotime, Shanghai, China) in TBST for 1 h. The membranes were immunoblotted with primary antibodies at 4 °C overnight, followed by incubation with HRP-conjugated secondary antibodies (25 °C, 1 h). Signal detection was performed using an ECL reagent and a Tanon gel imaging system (Tanon, Shanghai, China).

### 4.15. Real-Time Quantitative Polymerase Chain Reaction (RT-qPCR)

Following RNA extraction and reverse transcription from H9c2 cells, qPCR was conducted using a standardized protocol: 95 °C for 30 s, then 40 cycles of 95 °C for 10 s and 60 °C for 30 s. Gene expression was normalized to *GAPDH* and analyzed via the 2^(−ΔΔCt) method. *Nrf2* primers (Forward: 5′-TGAGAGCTCAGTCTTCACCACC-3′; Reverse: 5′-AATCAGTCATGGCCGTCTCC-3′) and *GAPDH* control primers (B661204) were supplied by Sangon Biotech (Shanghai) Co., Ltd. (Sangon Biotech, Shanghai, China).

### 4.16. Statistical Analysis

The data are presented as mean ± standard deviation (mean ± SD) for the individual groups. Statistical analysis was performed with GraphPad Prism 9.0. (GraphPad Software, San Diego, CA, USA) Statistical significance was determined by one-way analysis of variance (ANOVA) followed by comparison of different test groups using the Tukey test. Statistical significance was defined as *p* < 0.05.

## 5. Conclusions

Our study highlights the pivotal role of LBP in mitigating pyroptosis and MIRI in rats and H9c2 cells via modulation of the Nrf2/NLRP3 signaling pathway. By demonstrating that LBP can overexpress Nrf2 to inhibit NLRP3 and pyroptosis, and enhance cell viability, we provide a foundation for the potential treatment of MIRI with LBP.

## Data Availability

Data will be made available on request.

## References

[1] Zhang S., Yan F., Luan F., Chai Y., Li N., Wang Y.W., Chen Z.L., Xu D.Q., Tang Y.P. (2024). The pathological mechanisms and potential therapeutic drugs for myocardial ischemia reperfusion injury. Phytomedicine.

[2] Xiao H., Zhang M., Wu H., Wu J., Hu X., Pei X., Li D., Zhao L., Hua Q., Meng B. (2022). CIRKIL Exacerbates Cardiac Ischemia/Reperfusion Injury by Interacting with Ku70. Circ. Res..

[3] Gao Y., Wei Y., Wang Y., Gao F., Chen Z. (2017). *Lycium barbarum*: A Traditional Chinese Herb and A Promising Anti-Aging Agent. Aging Dis..

[4] Zhang Z., Liu H., Yu B., Tao H., Li J., Wu Z., Liu G., Yuan C., Guo L., Cui B. (2020). *Lycium barbarum* polysaccharide attenuates myocardial injury in high-fat diet-fed mice through manipulating the gut microbiome and fecal metabolome. Food Res. Int..

[5] Liu W.J., Jiang H.F., Rehman F.U., Zhang J.W., Chang Y., Jing L., Zhang J.Z. (2017). *Lycium barbarum* Polysaccharides Decrease Hyperglycemia-Aggravated Ischemic Brain Injury through Maintaining Mitochondrial Fission and Fusion Balance. Int. J. Biol. Sci..

[6] Lu S.P., Zhao P.T. (2010). Chemical characterization of *Lycium barbarum* polysaccharides and their reducing myocardial injury in ischemia/reperfusion of rat heart. Int. J. Biol. Macromol..

[7] Li Q., Zhang Z., Li H., Pan X., Chen S., Cui Z., Ma J., Zhou Z., Xing B. (2019). *Lycium barbarum* polysaccharides protects H9c2 cells from hypoxia-induced injury by down-regulation of miR-122. Biomed. Pharmacother..

[8] Song J., Luo X., Xiong L., Li S., Chen Y., Liu W., Yuan Y., Ma Y., Bian J., Liu Z. (2025). *Lycium barbarum* polysaccharide alleviate cadmium-induced mitochondrial dysfunction mediated pyroptosis in duck liver. Int. J. Biol. Macromol..

[9] Wu C., Zhang X.C., Chen L.R., Huang H.Z., Wu W.Y., Wang Y., Li G. (2024). Pyroptosis and mitochondrial function participated in miR-654-3p-protected against myocardial infarction. Cell Death Dis..

[10] Wang K., Sun Y., Zhu K., Liu Y., Zheng X., Yang Z., Man F., Huang L., Zhu Z., Huang Q. (2024). Anti-pyroptosis biomimetic nanoplatform loading puerarin for myocardial infarction repair: From drug discovery to drug delivery. Biomaterials.

[11] Ahmed S.M., Luo L., Namani A., Wang X.J., Tang X. (2017). Nrf2 signaling pathway: Pivotal roles in inflammation. Biochim. Biophys. Acta Mol. Basis Dis..

[12] Yao D., Bao L., Wang S., Tan M., Xu Y., Wu T., Zhang Z., Gong K. (2024). Isoliquiritigenin alleviates myocardial ischemia-reperfusion injury by regulating the Nrf2/HO-1/SLC7a11/GPX4 axis in mice. Free Radic. Biol. Med..

[13] Wang X., Wu S., Jiang Y., Yuan Z., Liu J., Jing S., Liu J., Sun J., Wang C., Wang D. (2025). Anwulignan alleviates IRI by the activation of Nrf2/HO-1 signaling pathway and inhibiting NLRP3-caspase-1-GSDMD-mediated pyroptosis in rats. Tissue Cell.

[14] Li J., Wang X., Zhang Y., Wei M., Qi J., Liu D., Wu R., Chen Q., Huang J. (2025). Ginsenoside Rg1 alleviates PCPA-induced insomnia by inhibiting NLRP3 inflammasome activation and pyroptosis through the Nrf2/HO-1 pathway in mice. Psychopharmacology.

[15] Duan Y., Li Q., Wu J., Zhou C., Liu X., Yue J., Chen X., Liu J., Zhang Q., Zhang Y. (2024). A detrimental role of endothelial S1PR2 in cardiac ischemia-reperfusion injury via modulating mitochondrial dysfunction, NLRP3 inflammasome activation, and pyroptosis. Redox Biol..

[16] Downey J.M., Omar B., Ooiwa H., McCord J. (1991). Superoxide dismutase therapy for myocardial ischemia. Free Radic. Res. Commun..

[17] Arroyo C.M., Kramer J.H., Dickens B.F., Weglicki W.B. (1987). Identification of free radicals in myocardial ischemia/reperfusion by spin trapping with nitrone DMPO. FEBS Lett..

[18] Przyklenk K., Kloner R.A. (1989). “Reperfusion injury” by oxygen-derived free radicals? Effect of superoxide dismutase plus catalase, given at the time of reperfusion, on myocardial infarct size, contractile function, coronary microvasculature, and regional myocardial blood flow. Circ. Res..

[19] Pinilla-González V., Rojas-Solé C., Gómez-Hevia F., González-Fernández T., Cereceda-Cornejo A., Chichiarelli S., Saso L., Rodrigo R. (2024). Tapping into Nature’s Arsenal: Harnessing the Potential of Natural Antioxidants for Human Health and Disease Prevention. Foods.

[20] Gao Y., Li H., Que Y., Chen W., Huang S.Y., Liu W., Ye X. (2024). *Lycium barbarum* polysaccharides (LBP) suppresses hypoxia/reoxygenation (H/R)-induced rat H9C2 cardiomyocytes pyroptosis via Nrf2/HO-1 signaling pathway. Int. J. Biol. Macromol..

[21] Zhang Q., Chen W., Zhao J., Xi W. (2016). Functional constituents and antioxidant activities of eight Chinese native goji genotypes. Food Chem..

[22] Baimakhanova B., Sadanov A., Bogoyavlenskiy A., Berezin V., Trenozhnikova L., Baimakhanova G., Ibraimov A., Serikbayeva E., Arystanov Z., Arystanova T. (2025). Exploring phytochemicals and their pharmacological applications from ethnomedicinal plants: A focus on *Lycium barbarum*, Solanacea. Heliyon.

[23] Yang X., Bai H., Cai W., Li J., Zhou Q., Wang Y., Han J., Zhu X., Dong M., Hu D. (2013). *Lycium barbarum* polysaccharides reduce intestinal ischemia/reperfusion injuries in rats. Chem. Biol. Interact..

[24] Liu J.J., Zhao G.X., He L.L., Wang Z., Zibrila A.I., Niu B.C., Gong H.Y., Xu J.N., Soong L., Li C.F. (2021). *Lycium barbarum* polysaccharides inhibit ischemia/reperfusion-induced myocardial injury via the Nrf2 antioxidant pathway. Toxicol. Rep..

[25] He W.T., Wan H., Hu L., Chen P., Wang X., Huang Z., Yang Z.H., Zhong C.Q., Han J. (2015). Gasdermin D is an executor of pyroptosis and required for interleukin-1β secretion. Cell Res..

[26] Wang L., Liu J., Wang Z., Qian X., Zhao Y., Wang Q., Dai N., Xie Y., Zeng W., Yang W. (2023). Dexmedetomidine abates myocardial ischemia reperfusion injury through inhibition of pyroptosis via regulation of miR-665/MEF2D/Nrf2 axis. Biomed. Pharmacother..

[27] Jain R., Vora L., Nathiya D., Khatri D.K. (2025). Nrf2-Keap1 Pathway and NLRP3 Inflammasome in Parkinson’s Disease: Mechanistic Crosstalk and Therapeutic Implications. Mol. Neurobiol..

[28] Liu Z., Deng P., Liu S., Bian Y., Xu Y., Zhang Q., Wang H., Pi J. (2023). Is Nuclear Factor Erythroid 2-Related Factor 2 a Target for the Intervention of Cytokine Storms?. Antioxidants.

[29] Zheng F., Yan L., Ren F., Cai W., Lan Y., Chen H., Chen Q., Weng G. (2025). Mechanism of Hypoxia-Induced HMGB1 Regulating NLRP3 Inflammasome/Caspase-1 Pathway-Mediated Pyroptosis in Myocardial Ischemia Reperfusion Injury Through the Nrf2/HO-1 Pathway. CNS Neurosci. Ther..

[30] Khounphinith E., Zhou Y., Yi Z., Li T., Li L. (2025). Vericiguat reduces pyroptosis in rats with coronary microembolization by inhibiting the AMPK/Nrf2/NLRP3 signaling pathway. Korean J. Physiol. Pharmacol..

